# Role of MMP-2(-1306 C/T) and TIMP-2(-418G/C) Polymorphism in Chinese Han Patients with Acne Vulgaris

**DOI:** 10.1155/2019/2364581

**Published:** 2019-03-27

**Authors:** Ruixue Gao, Heling Yu, Qian Zhao, Suhong Wang, Bingxue Bai

**Affiliations:** Department of Dermatology, The Second Affiliated Hospital of Harbin Medical University, Harbin, China

## Abstract

Acne is the most common chronic inflammatory skin diseases. Multiple factors, such as hormonal, environmental, immunological, and genetic factors, are thought to be involved in acne. However, genetic studies have yet to elucidate the full mechanism of acne. The aim of this study was to investigate the association of MMP-2 (-1306C/T) and TIMP-2 (-418G/C) polymorphisms with the risk of acne vulgaris in a Chinese Han population. We also analyzed the correlation of clinical parameters and family history in patients with acne vulgaris. This study included 251 acne patients and 121 age- and sex-matched healthy controls. Genomic DNA was extracted from peripheral blood, and genotyping was performed by PCR and DNA sequencing techniques. There is a significant correlation between the MMP-2 (-1306C/T) polymorphism and the acne vulgaris (P<0.001). Although no association was found between the TIMP-2 (-418G/C) polymorphism and the acne vulgaris, patients with the MMP-2 CT/TIMP-2 GG or GC allele are at higher risk of acne vulgaris. There is also a significant difference in the severity of the disease between acne vulgaris patients with and without family history (P<0.001). This study indicated that the MMP-2 (-1306C/T) polymorphism, in combination with the TIMP-2 (-418G/C) polymorphism, contributes to acne vulgaris susceptibility in the Chinese Han population.

## 1. Introduction

Acne is the most common chronic inflammatory skin disease in the follicular sebaceous glands in all races. The incidence rate of acne among the younger population is approximately 80-90% ranging from 16 to 20 years old, and acne can seriously affect their physical and mental health [1]. There are four main factors in the pathogenesis of acne, namely, hyperkeratinization, increased sebum production, colonization of skin bacteria (mainly Propionibacterium acnes (P. acnes)), and inflammation. Multiple factors, such as hormonal, environmental, immunological, and genetic factors, are thought to contribute to the incidence and development of acne [2, 3]. However, genetic studies have yet to elucidate the full mechanism of acne.

Matrix metalloproteinases (MMPs) and tissue inhibitors of metalloproteinases (TIMPs) play critical roles in the inflammatory response and matrix remodeling after inflammation [4, 5]. Several MMPs have been suggested to be involved in acne, and different levels of MMP expression could contribute to the development of different types of acne lesions [6–8]. Keratinocytes are an important source of MMPs in acne, and P. acnes can induce the expression of several MMPs, including MMP-1, -2, -3, -9, and -13 [9]. MMP-2 is a Zn^+2^-dependent endoproteinase, and its major natural inhibitor is TIMP-2. MMP-2 has been reported to be involved in disrupted integrity of sebaceous glands, and MMP-2 expression significantly decreased after treatment in a mouse model of P. acnes-induced inflammation, suggesting its role in acne [10–13]. However, the precise role of MMP-2 in acne remains unclear. Therefore, a better understanding of the correlation between phenotypes and genotypes of MMP-2 and TIMP-2 in patients with acne may help to further elucidate disease mechanisms.

Interestingly, in addition to its well-recognized function of degrading extracellular matrices such as collagens, laminin, and proteoglycans, recent studies highlight the involvement of MMP-2 in inflammation and lipid regulation [14, 15]. Decreased MMP-2 activity may lead to the production of cytokines such as IL-1*β*, Pro-IL-1*β*, TGF-*β*, latent TNF-*α*, and elevated triglycerides [16–19], which are the key modulators in acne.

MMP-2 activity can be regulated by several conditions such as other MMPs (e.g., MMP-3, MMP-9, and MMP-14), MMP inhibitors (e.g., TIMP-2 and RECK), and gene polymorphisms. Polymorphisms are common in the mmp2 and timp2 genes. Single functional nucleotide polymorphisms have been identified in the promoters of MMP-2 (-1306C/T) and TIMP-2 (-418G/C), which abolish the Sp1-binding site and result in decreased transcriptional activity of the genes [20, 21]. Previous studies have shown that the MMP-2 (-1306C/T) and TIMP-2 (-418G/C) polymorphisms are involved in the development of many cancers, such as breast, lung, and esophageal and colon cancer [22–25]. However, few studies have reported correlations of MMP-2 and TIMP-2 polymorphisms with acne.

The aims of this study were to investigate the association of MMP-2 (-1306C/T) and TIMP-2 (-418G/C) polymorphisms with the risk of acne vulgaris in a Chinese Han population and to explore the correlation of clinical phenotype and family history in acne vulgaris patients.

## 2. Materials and Methods

### 2.1. The Experimental Subjects

The study was conducted at the Second Affiliated Hospital of Harbin Medical University and was approved by the Ethical Committee of Harbin Medical University, and informed consent was obtained. Study participants comprised 251 patients with a diagnosis of acne aged 16-42 years.

The patients were enrolled according to the following criteria: (1) lesions on the face, chest, and back; (2) lack of other systemic diseases such as infection, cancer, and autoimmune diseases; and (3) lack of other skin diseases such as facial eczema, rosacea, and discoid lupus erythematosus.

All Chinese Han patients were diagnosed by at least two dermatologists. Severity of acne was evaluated according to Pillsbury's criteria: Grade 1, comedones and occasional small cysts confined to the face; Grade 2, comedones with occasional pustules and small cysts confined to the face; Grade 3, many comedones and small and large inflammatory papules and pustules, more extensive but confined to the face; and Grade 4, many comedones and deep lesions tending to coalesce and canalize, and involving the face and the upper aspects of the trunk. The subjects were categorized into three groups: mild acne (Grade 1) (43), moderate acne (Grade 2 and 3) (115), and severe acne (Grade 4) (93).

Of these patients, there were 141 subjects with family history of acne and 110 cases without family history of acne. Another 121 age- and gender-matched subjects with no acne served as controls. None of the participants had any systemic diseases. Informed consent was obtained from all subjects.

The relevant data collected included age, gender, weight, blood pressure, age of onset, type and severity of acne, duration of acne, and family history of acne. All participants were asked to contact their first- and second-degree relatives about their acne history. The first-degree relatives include the parents, siblings, and children of the patient. The second-degree relatives include grandparents, uncles, aunts, nieces, and nephews. A patient is considered to have a positive family history of acne, if at least one of the first- or second-degree relatives has or ever had acne.

### 2.2. Methods

#### 2.2.1. DNA Extraction and Genotyping

Genomic DNA was extracted from the peripheral blood of subjects using a Genomic DNA Purification Kit (Thermo Fisher Scientific) according to the manufacturer's instructions.

The SNPs of MMP-2 C1306T were genotyped by polymerase chain reaction (PCR). The following primers were used: MMP-2 forward, 5'-CTTCCTAGGCTGGTCCTTACTGA-3'; MMP-2 reverse, 5'-CTGAGACCTGAAGAGCTAAAGAGCT-3'; TIMP-2 forward, 5'-CGTCTCTTGTTGGCTGGTCA-3'; TIMP-2 reverse, 5'-CCTTCAGCTCGACTCTGGAG-3'. PCR products were then separated by 1.5% agarose gel electrophoresis. *β*-Actin is used as internal control. The purified PCR products were sequenced using an ABI377 automatic sequencer to identify the variants. To ensure the quality of the sequencing, 10% of the patient group and the control group, respectively, were randomly selected and genotyped twice. The reproducibility was 100%.

#### 2.2.2. Statistical Analysis

Statistical analysis was performed using SPSS 22.0 statistical software. The Mann-Whitney U test was used if the data did not meet a normal distribution. We first examined whether the genotype distribution in each group was in accordance with the Hardy-Weinberg equilibrium (HWE) by Pearson's X2 test. The association between acne and MMP-2/TIMP-2 polymorphisms was estimated by odds ratios (OR) and 95% confidence intervals (CI). The genotype distribution and allele frequency between the acne and the control groups were compared using the x2 test, and P <0.05 was considered statistically significant.

## 3. Results

### 3.1. General Clinical Data

The baseline characteristics of the subjects are summarized in Tables 1 and 2. The mean age of the 251 acne patients was 22.96 ± 5.42 years, and that of 121 controls was 23.28 ± 4.25 years. The analysis of the clinical characteristics, including age, gender, and BMI, showed no statistically significant differences between the two groups.

### 3.2. Association between MMP-2(-1306C/T) Polymorphism and Acne

The genotypes and allele frequencies of MMP-2 (-1306C/T) were compared between acne patients and controls (Table 3). The distribution of the analyzed MMP genotypes and allele frequencies followed the Hardy-Weinberg equilibrium. For acne, CC, CT, and TT genotypes were observed in 67.7%, 31.1%, and 1.2% of the patients and in 88.4%, 11.6%, and 0% of the controls, respectively. The CT + TT genotypes were significantly more prevalent in patients with acne vulgaris than in normal controls (P<0.001, OR=0.275). There was a significant difference in the T allele frequency between patients with acne and normal controls (P<0.001, OR=0.306) (Table 3).

**Figure 1:**
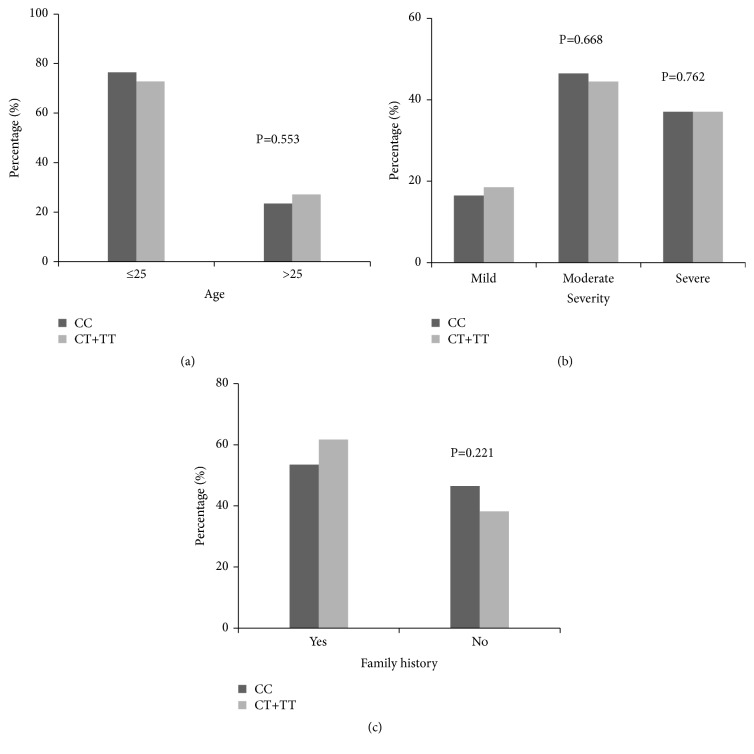
*Correlation between MMP-2 genotypes and clinical parameters*. The relationship between MMP-2 (-1306C/T) genotypes and clinical parameters was evaluated in patients with acne. There was no significant difference between the C and the T allele distributions in acne patients according to clinical parameters, such as age (a), severity (b), and family history (c) (P >0.05 for each). Correlation between MMP-2 (-1306C/T) genotypes and clinical parameters in acne.

Next, we evaluated the relationship between MMP-2 (-1306C/T) genotypes and clinical parameters in acne. As shown in Figure 1, there was no significant difference between the C and the T allele distributions in acne patients according to clinical parameters, such as age, severity, and family history (P>0.05 for each).

### 3.3. Association between TIMP-2(-418G/C) Polymorphism and Acne

There were no significant differences in the genotype distribution of TIMP-2 (-418G/C) between acne patients and normal controls (GG, GC, and CC genotype distributions as follows: 68.1%, 27.8%, and 4.8% in acne patients versus 73.6%, 23.9%, and 2.5% in normal controls) (Table 3). Although the CC genotype distribution was two times higher in acne vulgaris than in controls, no statistically significant correlation was found. Furthermore, no significant difference in the C allele frequency was found between patients with acne and normal controls.

Next, we investigated the correlation between TIMP-2 (-418G/C) genotypes and clinical parameters. There was no significant difference between G and C allele distribution with severity and family history in acne patients (P>0.05).

### 3.4. Interaction between MMP-2 and TIMP-2 Polymorphisms

**Figure 2:**
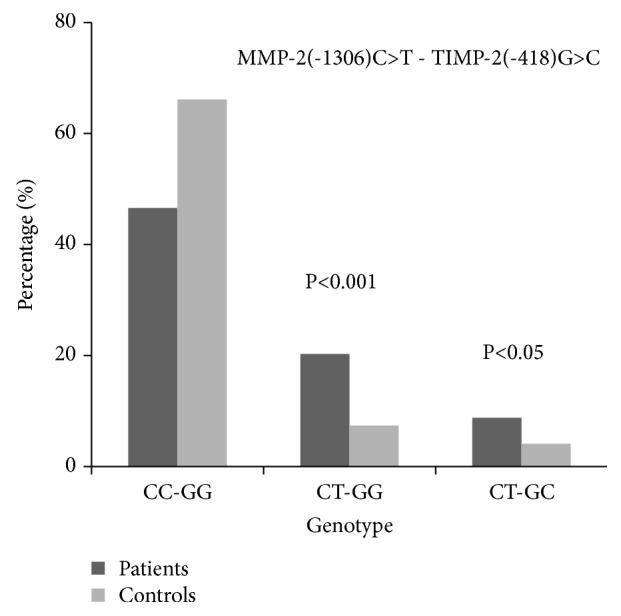
*Gene-gene interaction of MMP-2 and TIMP-2 in acne patients and healthy controls*. The combined effects of MMP-2 (-1306) C>T and TIMP-2 (-418) G>C polymorphisms were analyzed by gene interaction analysis. CT-GG and CT-GC genotype in patients with acne vulgaris were significantly higher than those in normal controls (p<0.001, OR=0.258; p <0.05, OR=0.332). MMP-2 and TIMP-2 gene polymorphism in acne.

The combined effects of MMP-2 (-1306C/T) and TIMP-2 (-418G/C) were analyzed by gene interaction analysis. CT-GG and CT-GC genotypes in patients with acne vulgaris were significantly higher than those in normal controls (P<0.001, OR=0.258; P<0.05, OR=0.332) (Figure 2). The TT-CC and TT-GC genotypes were not found in either the patient group or the control group. The other possible combinations did not show any significant association.

### 3.5. Correlation between Family History and Severity of Acne Vulgaris

56.2% patients had a family history of acne while 33.9% of the control group had a family history of acne. There was a statistical difference between the two groups (P < 0.001, OR = 2.036). Among patients with a family history of acne, 9.2% had mild acne and 90.8% had moderate to severe acne. Among patients without a family history, 27.3% had mild acne and 72.7% had moderate to severe acne. There was a significant difference in the severity of the disease between acne vulgaris patients with and without family history (P < 0.001, OR =3.692) (Table 4). Moreover, patients with a family history of acne were more likely to have cysts and nodules. There were no significant differences in gender distribution and age of onset between patients with and without family history (P>0.05) (Table 4).

## 4. Discussion

MMPs and TIMPs play an important role in inflammation and reduced integrity and remodeling of sebaceous glands in acne. Therefore, the balance between MMPs and TIMPs is vital in the development of acne. However, genetic studies and biomolecular tests have yet to elucidate the mechanism of acne.

This study shows that there was a significant correlation between the MMP-2 (-1306C/T) polymorphism and the acne in the Chinese Han population (P<0.001). Although no association was found between the TIMP-2 (-418G/C) polymorphism and the acne vulgaris, patients with MMP-2 CT/TIMP-2 GG or GC allele are at higher risk of acne vulgaris. MMP-2 is secreted as proMMP-2 and pro-MMP-2, TIMP-1, and TIMP-2 are subtly balanced under normal conditions. This finding suggests that interruption of the balance of MMP-2/TIMP-2 in gene expression by polymorphisms is related to acne vulgaris. The association of the MMP-2 (-1306C/T) and TIMP-2 (-418G/C) polymorphisms with the incidence and development of many cancers has been widely reported. However, there are very few reports about their association with acne vulgaris. Yaykasli et al. reported that MMP-2 (-1306C/T) and TIMP-2 (-418G/C) polymorphisms are not associated with acne vulgaris in the Turkish population, although expression of the TIMP-2 -418CC genotype was twice as high in the acne group as in the normal control group [26]. Our study is consistent with the results of TIMP-2 (-418G/C) in this report, but we found that patients with the MMP-2 (-1306C/T) polymorphism and with the MMP-2 CT/TIMP-2 GG or GC allele are at higher risk of acne vulgaris. The discrepancy might be due to different genetic backgrounds, smaller sample sizes, and lack of investigation of the combined effect of the two polymorphisms in the latter study.

Two functional nucleotide polymorphisms in the promoter of the MMP-2 gene (-1306C/T) and the TIMP-2 gene (-418G/C) were reported previously [21, 22]. The transitions of C→T in the MMP-2 promoter and G→C in the TIMP-2 promoter result in decreased transcriptional activity of both genes. In our study, a significantly higher percent of subjects carried the T allele in the acne vulgaris group than in the control group. Moreover, patients with the MMP-2 CT/TIMP-2 GG genotype were at higher risk of acne, suggesting that MMP-2 gene activity might be downregulated in acne vulgaris.

MMP-2 is now recognized to cleave a large number of non-matrix substrates, including IGFBPs, IL-1*β*, pro-IL-1*β*, latent TGF-*β*, and latent TNF-*α*, which are the main factors in acne inflammation [16, 18, 19]. Recent studies have suggested that if MMP-2 activity falls below baseline, there is an increase in proinflammatory cytokine production that results in system inflammation [14, 15]. Furthermore, MMP-2 deficiency results in lipid dysregulation, such as reduced levels of liver X receptor (LXR)-*α* and increased hepatic expression of triglycerides [17]. These molecules are also reported to be dysregulated in acne [27, 28]. Our study suggests that MMP-2 CT/TIMP-2 GG genotype may correspond to decreased activity of MMP-2, which may lead to the high expression of IL-1a and *β*, TNF-*α*, and triglycerides observed in acne patients. The latter further promote inflammation and lipid dysregulation in acne. Further studies are needed to explore the correlation between MMP-2 activity and expression of IL-1a and *β*, TNF-*α*, and triglycerides in acne.

In this study, we also investigated whether family history of acne was associated with various phenotypic features of acne vulgaris, including age of onset, sex, and clinical stage. Patients with familial history of acne had a significant higher prevalence of acne vulgaris than those of normal controls. We also note that patients with a positive family history present more severe outcomes. There was a significant difference in the severity of the disease between acne vulgaris patients with and without family history, indicating a genetic predisposition to acne vulgaris.

Our study has some limitations. Studies have shown that multiple SNPs can interact independently, collectively, or individually with each SNP, thus affecting the onset of disease. Therefore, multiple alleles or genes work together, not just a single gene that causes an increased risk of acne. In this study, a higher percentage of subjects had the MMP-2 CT/TIMP-2 GC genotype in the acne group than in the control group, suggesting that other polymorphisms might interact collectively in MMP-2 and TIMP-2 activity in acne vulgaris. Large number of samples and more biomolecular techniques are needed to analyze multiple gene interactions as well as to better predict the incidence of acne in future studies. In addition, more work is required to confirm these findings at the protein level.

In summary, this study suggests that the MMP-2 (-1306C/T) polymorphism in combination with the TIMP-2 (-418G/C) polymorphism are associated with an increased risk of acne in the Chinese Han population. The effects of MMP-2 (-1306C/T) and TIMP-2 (-418 G/C) polymorphisms on MMP-2 gene activity and the risk of acne vulgaris deserve further investigation.

## Figures and Tables

**Table 1:** Baseline characteristics of the subjects.

	Number	Gender (male/female)	Age (years)	BMI (kg/m^2^)
Controls	121	43/78	23.28 ± 4.25	19.96 ± 2.10
Acne	251	94/157	22.96 ± 5.42	20.18 ± 2.37
Total	372	137/253	23.37 ± 5.01	20.11 ± 2.28
P			0.102	0.343

Results are given as mean ± standard deviation. BMI: body mass index.

**Table 2:** Classification of acne patients.

	Acne type			Family history of acne	
	mild	moderate	severe	yes	no
Number	43	115	93	141	110

**Table 3:** Genotype frequencies of MMP-2 (-1306C/T) and TIMP-2 (-418G/C) polymorphism in acne patients and controls.

Genes	Controls	Patients	OR (95%CI)	*P*
	n=121 (%)	n=251 (%)		
MMP-2 (-1306) C>T				
CC	107 (84.4)	170 (67.7)	Reference	
CT	14 (11.6)	78 (31.1)	0.285 (0.154-0.529)	<0.001
TT	0 (0)	3 (1.2)	0.614 (0.559-0.674)	0.171
CT+TT	14 (11.6)	81 (32.3)	0.275 (0.148-0.509)	<0.001
Allele				
C	228 (94.2)	418 (83.2)	Reference	
T	14 (5.8)	84 (16.7)	0.306 (0.170-0.550)	<0.001
TIMP-2 (-418) G>C				
GG	89 (73.6)	171 (68.1)	Reference	
GC	29 (23.9)	68 (27.1)	1.220 (0.737-2.021)	0.439
CC	3 (2.5)	12 (4.8)	2.082 (0.573-7.569)	0.256
GC+CC	32 (26.4)	80 (31.9)	1.301 (0.802-2.110)	0.285
Allele				
G	207 (85.5)	410 (81.7)	Reference	
C	35 (14.5)	92 (18.3)	1.327 (0.869-2.027)	0.189

OR: odds ratio adjusted by acne patients and controls; CI: confidence interval.

**Table 4:** Correlation between family history and severity of acne vulgaris.

	Family history			
	Yes (%)	No (%)	OR (95%CI)	*P*
Group				
Controls	41 (33.9)	80 (66.1)	Reference	
Patients	141 (56.2)	110 (43.8)	2.036 (1.032-4.016)	<0.001
Patients Gender				
Male	47 (33.3)	47 (42.7)	Reference	
Female	94 (66.7)	63 (57.3)	0.670 (0.400-1.122)	0.127
Age of onset	18.60±5.30	19.54±6.04		0.194
Severity				
Mild	13 (9.2)	30 (27.3)	Reference	
Moderate	69 (48.9)	48 (43.6)		
Severe	59 (41.8)	32 (29.1)		<0.001
Moderate+Severe	128 (90.8)	80 (72.7)	3.692 (1.819-7.497)	<0.001

OR: odds ratio. CI: confidence interval.

## Data Availability

The data used to support the findings of this study are available from the corresponding author upon request.

## References

[1] Tan J. K. L., Bhate K. (2015). A global perspective on the epidemiology of acne. *British Journal of Dermatology*.

[2] Mwanthi M., Zaenglein A. L. (2018). Update in the management of acne in adolescence. *Current Opinion in Pediatrics*.

[3] Carlavan I., Bertino B., Rivier M. (2018). Atrophic scar formation in patients with acne involves long-acting immune responses with plasma cells and alteration of sebaceous glands. *British Journal of Dermatology*.

[4] Fingleton B. (2017). Matrix metalloproteinases as regulators of inflammatory processes. *Biochimica et Biophysica Acta (BBA) - Molecular Cell Research*.

[5] Cui N., Hu M., Khalil R. A. (2017). Biochemical and biological attributes of matrix metalloproteinases. *Progress in Molecular Biology and Translational Science*.

[6] Lee W., Jung H., Lim H., Jang Y., Lee S., Kim D. (2013). Serial sections of atrophic acne scars help in the interpretation of microscopic findings and the selection of good therapeutic modalities. *Journal of the European Academy of Dermatology and Venereology*.

[7] Saint-Jean M., Khammari A., Jasson F., Nguyen J. M., DrΘno B. (2016). Different cutaneous innate immunity profiles in with and without atrophic scars. *European Journal of Dermatology*.

[8] Ozkanli S., Karadag A. S., Ozlu E. (2016). A comparative study of MMP-1, MMP-2, and TNF-*α* expression in different acne vulgaris lesions. *International Journal of Dermatology*.

[9] Lee S. E., Kim J.-M., Jeong S. K. (2010). Protease-activated receptor-2 mediates the expression of inflammatory cytokines, antimicrobial peptides, and matrix metalloproteinases in keratinocytes in response to Propionibacterium acnes. *Archives of Dermatological Research*.

[10] Choi J. Y., Piao M. S., Lee J. B., Oh J. S., Kim I. G., Lee S. C. (2008). Propionibacterium acnes stimulates pro-matrix metalloproteinase-2 expression through tumor necrosis factor-alpha in human dermal fibroblasts. *Journal of Investigative Dermatology*.

[11] Sato T., Kurihara H., Akimoto N., Noguchi N., Sasatsu M., Ito A. (2011). Augmentation of gene expression and production of promatrix metalloproteinase 2 by Propionibacterium acnes-derived factors in hamster sebocytes and dermal fibroblasts: A possible mechanism for acne scarring. *Biological & Pharmaceutical Bulletin*.

[12] Han R., Blencke H.-M., Cheng H., Li C. (2018). The antimicrobial effect of CEN1HC-Br against Propionibacterium acnes and its therapeutic and anti-inflammatory effects on acne vulgaris. *Peptides*.

[13] Fan X., Xing Y.-Z., Liu L.-H. (2013). Effects of 420-nm intense pulsed light in an acne animal model. *Journal of the European Academy of Dermatology and Venereology*.

[14] Hardy E., Hardy-Sossa A., Fernandez-Patron C. (2018). MMP-2: is bad as in the cardiovascular system?. *American Journal of Physiology-Heart and Circulatory Physiology*.

[15] Rodríguez D., Morrison C. J., Overall C. M. (2010). Matrix metalloproteinases: what do they not do? New substrates and biological roles identified by murine models and proteomics. *Biochimica et Biophysica Acta*.

[16] Ito A., Mukaiyama A., Itoh Y. (1996). Degradation of 1 beta by matrix metalloproteinases. *Journal of Biological Chemistry*.

[17] Fernandez-Patron C., Kassiri Z., Leung D. (2016). Modulation of systemic metabolism by MMP-2: from MMP-2 deficiency in Mice to MMP-2 deficiency in patients. *Comprehensive Physiology*.

[18] Dallas S. L., Rosser J. L., Mundy G. R., Bonewald L. F. (2002). Proteolysis of latent transforming growth factor-beta (TGF-beta )-binding protein-1 by osteoclasts. a cellular mechanism for release of TGF-beta from bone matrix. *Journal of Biological Chemistry*.

[19] De Groef L., Salinas-Navarro M., Van Imschoot G. (2015). Decreased TNF levels and improved retinal ganglion cell survival in MMP-2 null mice suggest a role for MMP-2 as TNF sheddase. *Mediators of Inflammation*.

[20] Price S. J., Greaves D. R., Watkins H. (2001). Identification of novel, functional genetic variants in the human matrix metalloproteinase-2 gene. role of Sp1 in allele-specific transcriptional regulation. *The Journal of Biological Chemistry*.

[21] De Clerck Y. A., Darville M. I., Eeckhout Y., Rousseau G. G. (1994). Characterization of the promoter of the gene encoding human tissue inhibitor of metalloproteinases-2 (TIMP-2). *Gene*.

[22] Hernandez‐Anzaldo S., Berry E., Brglez V. (2015). Identification of a novel heart–liver axis: matrix metalloproteinase‐2 negatively regulates cardiac secreted phospholipase A2 to modulate lipid metabolism and inflammation in the liver. *Journal of the American Heart Association*.

[23] Banday M. Z., Sameer A. S., Mir A. H., Mokhdomi T. A., Chowdri N. A., Haq E. (2016). Matrix metalloproteinase (MMP) -2, -7 and -9 promoter polymorphisms in colorectal cancer in ethnic Kashmiri population—a case–control study and a mini review. *Gene*.

[24] Zagouri F., Sergentanis T. N., Gazouli M. (2013). MMP-2 -1306C > T polymorphism in breast cancer: a case-control study in a South European population. *Molecular Biology Reports*.

[25] McColgan P., Sharma P. (2009). Polymorphisms of matrix metalloproteinases 1, 2, 3 and 9 and susceptibility to lung, breast and colorectal cancer in over 30,000 subjects. *International Journal of Cancer*.

[26] Yaykasli K. O., Turan H., Kaya E., Hatipoglu O. F. (2013). Polymorphisms in the promoters of MMP-2 and TIMP-2 genes in patients with acne vulgaris. *International Journal of Clinical and Experimental Medicine*.

[27] Bakry O. A., El Farargy S. M., El Kady N. N. E. D., Dawy H. F. A. (2017). Immunohistochemical expression of cyclo-oxygenase 2 and Liver X receptor-*α* in Acne Vulgaris. *Immunohistochemical Expression of Cyclo-oxygenase*.

[28] Jiang H., Li C. Y., Zhou L. (2015). Acne patients frequently associated with abnormal plasma lipid profile. *The Journal of Dermatology*.

